# Electrokinetic Properties of the Pristine and Oxidized MWCNT Depending on the Electrolyte Type and Concentration

**DOI:** 10.1186/s11671-016-1367-z

**Published:** 2016-03-24

**Authors:** Ewa Skwarek, Yuliia Bolbukh, Valentyn Tertykh, Władysław Janusz

**Affiliations:** Maria Curie-Sklodowska University, Sq. Maria Curie-Sklodowska 2, Lublin, 20-031 Poland; Chuiko Institute of Surface Chemistry of NAS Ukraine, 17 General Naumov Str, Kyiv, 03164 Ukraine

**Keywords:** Multiwalled carbon nanotubes, Monovalent electrolytes, Electrolyte concentration, Aqueous dispersion, Size distribution, Zeta potential, Surface charge, 81.07.De, 82.45.Rr, 82.45.Jn

## Abstract

Electrostatic stabilization is reduced in its efficiency in an electrolyte-containing environment. The effect of electrolyte concentration is mostly described as negative factor for dispersion stabilization. Usually, zeta potential and physical stability decrease at increasing electrolyte concentration. The purpose of the present study was to measure the surface properties of nanotubes in aqueous solution of monovalent electrolytes at different concentration. Characteristics such as size distribution, surface chemistry, surface charge, and dispersability in aqueous phase have been identified. Hydrodynamic size and zeta potential in aqueous multiwalled carbon nanotube (MWCNT) suspensions were determined at different pH with the desired concentrations of electrolyte of the cationic group (NaCl, KCl, CsCl) and the anionic group (NaClO_4_). The correlations between the response of the surface functionality of pristine and oxidized multiwalled carbon nanotubes and electrical double layer (EDL) forming at different ionic environments in the vicinity of a nanotube surface were determined. The nanotube dispersion stabilization was found to be more affected by ion size and pH medium then electrolyte concentration. The data obtained confirms the predominant role of surface reactions. The most stable dispersion of nanotubes was achieved in KCl electrolyte solution at less negative charge of the surface.

## Background

Carbon nanomaterials have a large surface area and complex particle’s shape architecture. These qualities are successfully applying to develop new drug carriers, fillers, or nanodetectors [1]. At the same time, the utilization efficiency of such materials is caused by maintaining of the nanodispersity. An agglomeration and partially sedimentation of nanotubes in aqueous media is a well-known fact. The stability of the nanotube dispersion mostly depends on the uniformity of the boundary layer and nanotube morphology. The hydrophobic nature of defectless areas of their surface and the hydrophilicity of some types of defects in the nanotube boundary layer, namely carboxy- or phenolic groups [2, 3] which are formed during nanotube purification and washing, are competing during dispersing in the aqueous media. An increase in stability of the nanotube dispersion in water can be achieved through the surface charge via formation of the double electrical layer (EDL). Among other carbon materials, carbon nanotubes are characterized by the ability to accumulate charge, fast cycle of charge/discharge, and these properties are important for the development of devices operating on the principle of EDL. The rapid development of technology of preparation of ionistors based on application of nanotubes determines the relevance of studies of EDL formation on nanotubes with different functionality in the electrolyte medium. In addition, the behaviour of nanotube particles in electrokinetic experiments may provide information on the surface properties and the interactions among particles suspended in a liquid. The EDL formation phenomenon depends on the fact that a potential difference is developed when two phases are in contact. There are several ways to generate this effect in aqueous systems. The water dipoles may be oriented at the interface, thus creating a potential difference. Ions or excess electrons in one or both phases give rise to a nonuniform distribution of electric charges at the interface between the phases. Furthermore, in the development of a surface charge that exists on the solid surface, whether by ion adsorption from the liquid phase on the particle or ionization of groups, the surface acquires a potential with respect to the solution [4].

Another important question is what functional sites on the nanotube boundary layer have predominant effect on EDL formation and act as potential-determining ions (PDI). This term is usually reserved for those involved in whatever process is responsible for the particle charge. For example, most nanocarbon microspheres or nanoparticles are charged because they have carboxylate groups on the surface; ionization of these groups leads to the charge, so H+ is a PDI on this surface. Carbon nanotubes besides carboxyl groups contain lactone, phenylic, and carbonyl groups, as well as structural defects with ester or ether groups in rings on nanotube caps and walls [5], amorphous debris with different structure on the walls [6], and polycyclic aromatic substances, classified as fulvic acids [7]. In the work [8], it has been shown that the amount of oxygen-containing groups at the OH-terminated surface does not show an apparent change in the absorption ability of carbon nanotubes. On the nanotube surface, an influence of oxygen and hydrogen atoms to cancel out each other gives a slightly higher charge. In the chemisorption or oxidation process, the formation of new chemical bonds on nanotubes leads to the change of surface dipoles. The distinction between specifically adsorbed and potential determining ions is often vague, particularly in those systems in which the surface chemistry is not fully understood.

As outlined in literature for colloidal particles, the zeta potential and physical stability decrease at increasing electrolyte concentration. Also, the decrease in zeta potential was more pronounced when moving from Na^+^ to Mg^2+^ and to Al^3+^. In the case of similar valence of cation, the zeta potential decreases in line: Na^+^ < К^+^ < Cs^+^ for hydrophobic organic materials (polymers, nanocrystals, carbon nanoparticles) and metal oxides at low pH_pzc_. For metal oxides at high pH_pzc_, the inverse sequence was observed. The inverse sequence may be a result of making or breaking of water structure near the surface of metal oxide in aqueous electrolyte and/or preferential interaction between surface groups and counter ion as a result of “similar seeks for similar” [9].

Although all ions tend to reduce the zeta potential, the Cs^+^ ions were the most effective, in accordance with the Schultz and Hardy rule (Van Olphen, 1977). The Cs^+^ ions have small hydrated ionic radii, a small distance of the closest approach to hydrophobic surfaces and large polarizability compared to K^+^ and Na^+^. These properties allow Cs^+^ ions to approach the surface of the hydrophobic particles more readily and to become specifically adsorbed. Consequently, the electrical double layer of the particle was compressed. Therefore, the zeta potential of the particles with Cs^+^ electrolyte indicates more positive values than particles in NaCl or KCl electrolytes. The similar trend should be observed for anion with different size but with same valence. But, this is depending on native surface ability to ions adsorption.

### Background of the Study

A prerequisite to achieve an enhancement in the efficiency of the utilizing of carbon nanotubes in aqueous media is that the nanoparticles are finely dispersed. Preventing aggregation is necessary both for coating production and for applications of nanotubes as a carrier [10]. Characteristics such as size distribution, surface chemistry, surface charge, and dispersion in aqueous phase have been identified as essential in studying the environmental fate of MWCNTs. Such data is essential because the interactions of environmental and biological systems are dependent on them [11].

In terms of knowledge of the zeta potential depending on the nature and concentration of the electrolyte can be achieved to stabilize the dispersion of carbon nanotubes in an aqueous medium without the use of additional organic stabilizing compounds. The effect of electrolyte is mostly described as negative factor for nanoparticle’s dispersion stabilization: zeta potential and physical stability decrease at increasing the electrolyte concentration. But for dispersion of carbon nanotubes, which have charged surface groups and can be stabilized due to steric factor, the electrolyte presence should promote better dispersibility. The purpose of the present study was to measure the surface properties of carbon nanotubes in aqueous solution of monovalent electrolytes at different concentrations. This work is aimed to determine the correlation between the response of the surface functionality of pristine and oxidized multiwalled carbon nanotubes and EDL forming at different ionic environment in the vicinity of the nanotube surface. Optimum parameters of dispersion stabilizing were identified in a systematic screening based on zeta potential measurements.

## Methods

Investigation was performed with multiwalled carbon nanotubes (MWCNTin) produced in the Chuiko Institute of Surface Chemistry (Ukraine) using the catalytic vapour deposition method [12]. The carbon nanotubes have been functionalized, and the specific surface area of the pristine and functionalized nanotubes was measured using N_2_ ad-/desorption. For surface functionalization, nanotubes were oxidized with oxygen peroxide as described in [13]. Briefly, purified MWCNTs were dispersed in water, and then hydrogen peroxide was added to suspension. The mixture was heated at 80 °C under stirring for 47 h. The concentration of H_2_O_2_ was 30 %. The obtained oxidized nanotubes were filtered under vacuum and dried at 150 °C. The pristine and oxidized (MWCNTox) nanotubes were characterized by TEM spectroscopy, and the titrimetric method to evaluate the contents of the surface functional groups.

*Characterization of MWCNTs*. Surface morphology and MWCNT diameter were determined using high-resolution transmission electron microscopy (HR TEM, JEOL Jem-2100). Oxidation of MWCNTs was confirmed using FT-IR spectroscopy where vibrational peaks of C=O, C–O, and O–H bonds were detected in oxidized MWCNTs.

Hydrodynamic size and zeta potential in aqueous MWCNT suspensions were determined using dynamic light scattering (Malvern Zetasizer Nano series, NanoZS). The zeta-potential values were recorded using a Zetasizer (ZEN 3600, Malvern) and a Zetasizer nano ZS90 (Malvern) at room temperature as a function of pH (2–11.8) using a solution of electrolytes in water. The measurement itself is a particle electrophoresis; the particle velocity is determined via the Doppler shift of the laser light scattered by the moving particles. The field strength applied was 20 V/cm. The electrophoretic mobility was converted to the zeta potential in mV using the Helmholtz-Smoluchowski equation. At standard measuring conditions (room temperature of 25 °C, water), this equation can be simplified to the multiplication of the measured electrophoretic mobility (μm/cm per V/cm) by a factor of 12.8, yielding the ZP in mV. Before measuring the zeta potential and particle size, there were prepared the aqueous NaCl, KCl, CsCl, NaClO_4_, solutions of the concentration 10^−1^ mol/dm^3^. Then, it was ultrasonicated in a Sonicator XL2020 produced by Misonix for 3 min to achieve complete dispersion of the sediment. The obtained suspension was poured into Erlenmeyer flasks. In the next stage, the pH values of the suspension were established by dropping in 0.1 mol/dm^3^ and 0.01 mol/dm^3^ HCl or NaOH, CsOH, KOH solution.

### Preparation of MWCNT Suspensions of Various Ionic Strengths

A nanotube-water suspension of a given solid content (wt %) was prepared at different pH with the desired concentrations of electrolyte of the cationic group (NaCl, KCl, CsCl) and the anionic group (NaClO_4_). The pH of the suspension was adjusted using aqueous solutions of HCl and NaOH, CsOH, KOH; in preliminary tests, it was observed that the measured pH of the supernatant solution differed by only 0.2–0.4 from that of the original suspension. After conditioning, approximately 3 ml of suspension was transferred into the sample cell of an instrument for zeta potential and particle size distribution measurement. For measurement of surface charge, the suspensions with demanded content of solid phase were prepared at pH of 3. The measurement of the zeta potential, particle distribution, and the surface charge was performed in NaCl, KCl, CsCl, or NaClO_4_ solutions with concentration of each electrolyte of 0.1, 0.01, and 0.001 mol/l, respectively. For all tests, the nanotube concentration was maintained at 0.2 mg/ml. The suspensions were sonicated three times for 1 min and were shaken before measurements. For study, the sedimentation/flotation process selected suspensions were prepared without sonication. In this case, the measurement of the particle distribution and zeta potential was performed before and after sonication several times during 30 min.

The surface charge density at the multiwalled carbon nanotubes (MWCNTin)/electrolyte solution interface was determined by potentiometric titration of the suspension. The measurement was carried out in a thermostated Teflon vessel, under nitrogen atmosphere free of CO_2_, at 25 °C. Measurements of pH were carried out using a PHM 240 Radiometer Research pH metre with K401 as a glass electrode and G202B as a reference calomel electrode. Potentiometric titration was carried out with the use of the automatic burette Dosimat 665 (Metrohm). The whole experiment was controlled by computer software. Potentiometric titration, as well as electrokinetic measurements, was made at three different concentrations: 0.001, 0.01, and 0.1 mol/dm^3^ of NaCl, KCl, CsCl, and NaClO_4_ solutions. All the solutions were prepared with double distilled water. All the reagents used for experiments were of analytical grade purity.

## Results and Discussion

### Morphology Characteristics of Pristine and Oxidized MWCNTs

Parameters such as morphology, size distribution, surface area, surface chemistry, and agglomeration state as well as purity of the samples have considerable impact on the surface charge and the reactivity of carbon nanotubes [14]. It has been shown [8] that the charge on the surface of pristine nanotubes depends on the geometry of the nanotubes. Types and distribution of defects on the outer layers of multiwalled nanotubes determine the ability of the nanotubes to retain ions in the surface layer when forming electric double layer, and, consequently, a stability of nanotube dispersion in the aqueous electrolyte solution. Upon the nanotube oxidation, oxygen-containing groups are formed in the boundary layers, namely carbonyl, carboxyl, lactone, quinone, ether, and hydroxyl groups, the content and distribution of which depend on the oxidation method. It should be also noted that during the oxidation process, cleaning from amorphous part of the sample, the destruction of defects on outer nanotube layers, and in preferable case, the opening of ports that provides access to the inner cavity occur.

**Fig. 1:**
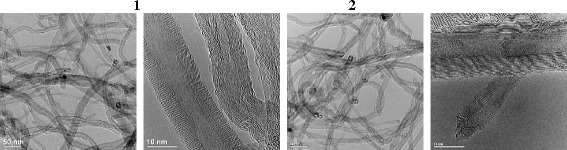
TEM images of pristine and oxidized MWCNTs. Photos show pristine (initial or as synthesized) multiwalled carbon nanotubes (MWCNTin) (*1*) and carbon nanotubes oxidized by treatment with hydrogen peroxide (MWCNTox) (*2*) at magnification of 50 and 10 nm

Table 1 shows the characteristics of a surface of the nanotubes before and after oxidation with the hydrogen peroxide solution. The TEM micrograph of pristine MWCNTs (Fig. 1) showed a mixture of catalyst residues and some protruding fibre-like structures. Images of oxidized nanotubes (MWCNTox) display that after oxidation, both cleaning the outer surface of carbon nanotubes from amorphous debris and the partial destruction of nanotubes walls and ports occur. Pristine nanotubes are about 10–20 nm in diameter with an amount of a layer of 7–20. After oxidation of the nanotubes, specific surface area increases due to both a partial thinning of nanotubes and ports opening. Also, pore size is increased to a mesoporous scale.

**Table 1:** Structural characteristic of pristine and oxidized multiwalled carbon nanotubes

Sample	Specific surface area (BJH), m^2^/g	Pore Volume (BJH), cm^3^/g	*D*_pores_ (BJH), Å	Average MWCNT diameter, nm	Number of layers	Concentration of oxygen-containing groups, mmol/g
MWCNTin	150	0.55	34	15-20	7-20	10.9
MWCNTox	240	2.03	148	10-15	5-15	21.9

The average diameter of MWCNTs was found to be 20–15 nm, and the oxidized MWCNTox had a diameter range of 15–10 nm (Figs. 1 and 4).

### Particle Size Distribution of the Pristine and Oxidized Nanotubes in Aqueous Suspension of Monovalent Electrolytes

The nanotubes tend to aggregate depending on the features of nanotube structure, the nature of functional groups, and the nature of the dispersion medium, that is why a careful selection of the measurement conditions is required.

Increasing the time of sonication can lead to increased aggregation due to occurrence of an electrostatic charge on the nanotube surface. On the other hand, the impact oscillations of different frequencies, as shown in [15], are capable to decrease a thickness of formed EDL due to occurrence of the potential difference between two points located at different distances from the radiation source, and a decrease in the thickness of the EDL is stronger at the higher oscillation frequency.

One should also consider the possibility of nanotubes crushing under the action of ultrasonic waves, especially after oxidation [16, 17]. Furthermore, unlike the dispersions of spherical particles, an important parameter of suspension stability is shape factors of multiwalled carbon nanotubes. A nanotube’s three-dimensional shape on the one hand can promote the stabilization of the dispersion in case of nanotubes expanded state with charge concentration on outside end, on the other hand, it can cause the suspension exfoliation due to multiplicity of contacts between the particles, and as a result, a discreteness of formation and distribution of the charge along the nanotubes surface, in inner and outer area of aggregates. As shown in [18], after cutting of the nanotubes, the stability of aqueous dispersions was increased, on the other hand, a decrease in particle size is marked increase in aggregation [19, 20]. Nonuniform distribution of the active sites with a concentration of charged groups on structural defects (bends, kinks, wall cavities) and on ends of nanotubes considerably complicates the stabilization of the dispersion.

It should be also noted that due to the aggregation of the nanotubes even at low concentrations, the formation of the charged layer can occur both on the outer boundary side and inside the units. Also, a diffusion of the electrolyte aqueous medium into tightly packed aggregates can be complicated by partial hydrophobicity of carbon nanotubes. In this case, the formation of a charged stabilizing layer will be realized mostly at the outer edge of aggregated nanotubes. It was noted earlier that the zeta potential of the pristine nanotubes under study changes from negative to positive with an increase in aggregate size [2]. During this accumulation of charge, a charge or discharge inside the unit and an extended in time response to changes in the ionic environment (for example, during titration) could be marked.

**Fig. 2:**
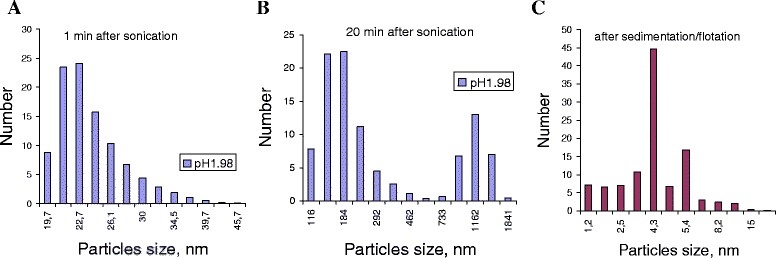
Particle size distribution in dispersions of pristine MWCNTs in solution of NaCl. Graphs show the particle size distributions in the dispersion of pristine MWCNTs (dispersion concentration 0.2 mg/ml) in NaCl solution (C = 0.1 mol/l) at pH = 1.89. The dispersion was prepared by sonication during 3 min. The measurements were performed at 1 min (**a**), 20 min (**b**), and 30 min (**c**) after sonication without disturbing of the dispersion

The stability of the dispersions after preparation was studied. The diagram (Fig. 2) shows the distribution of particles size (aggregates) for pristine nanotubes vs time after sonication.

The particle size distribution in dispersion of the pristine nanotubes in NaCl (C = 0.1 mol/l and pH 1.89) changes in time. It was found that nanotube agglomerates are formed in about 2–5 min after sonication and that the monodispersion with prevalent effective particle size of about 22 nm transforms into polydispersion with particle sizes of 180 and 1160 nm. Dispersion processes are followed by simultaneous sedimentation and flotation that implies a different density of nanotube agglomerates, and only particles with diameter close to 4.5 nm are found in the solution volume. Nevertheless, the dispersion returns to the monodisperse state (particle size of about 590 nm) after shaking.

### Particle Size Distribution of the Pristine and Oxidized Nanotubes in Aqueous Suspension at Different pH

**Fig. 3:**
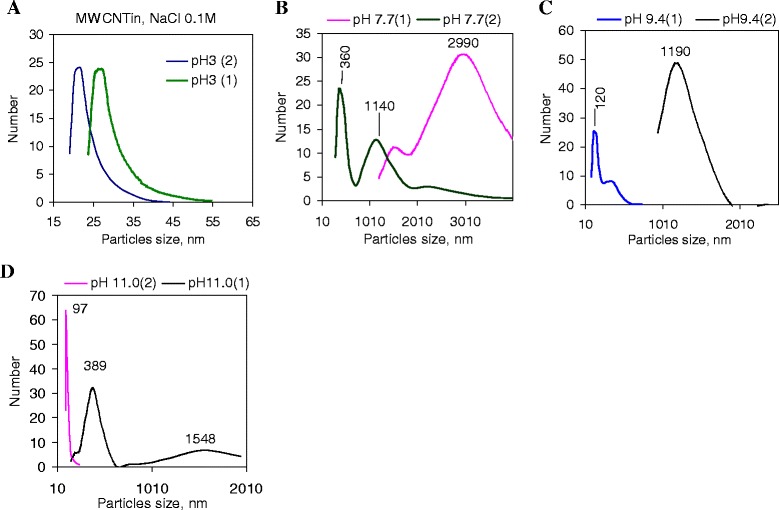
The particle size distribution in dispersions of pristine MWCNTs in NaCl solution at different pH. Graphs show the particle size distribution in dispersion of pristine carbon nanotubes (MWCNTin) (dispersion concentration of 0.2 mg/ml) in NaCl solution (C = 0.1 mol/l) at pH = 3.0 (**a**), pH = 7.7 (**b**), pH = 9.4 (**c**), and pH = 11.0 (**d**) before (*1*) and after sonication (*2*). Ultrasonic treatment was performed under ultrasound at 22 kHz during 3 min. The measurements were carried out in 1 min after sonication

Comparison of the results for series of pristine nanotube suspensions at NaCl concentration of 0.1 mol/l indicated that there was a significant difference between particle size distributions at different pH values. At pH = 3 for electrolyte solution, a hydrodynamic particle size in the pristine nanotube (MWCNTin) dispersion was stabilized (Fig. 3a) and changes were negligible before and after sonication. The high pH transition (pH 7–11) involves irreversible conformational changes resulting in significant increase in a hydrodynamic particle size (Fig. 3b–d, *curve 1*), and the suspension becomes polydisperse, which indicates an increase in the interaction between the particles and can be the result of the particle charge compensation, or changes of potential-determining ions in the structure of the EDL. The bimodal particle size distribution is maintained after sonication (Fig. 3b, c, *curve 2*). However, in dispersions under study (NaCl 0.1 M) in the range from pH of 7 to 11, the main mode in particle size distribution curve shifts toward lower hydrodynamic particle size, indicating increased repulsion between nanotube aggregates. According to the measurement of the particle size distribution at pH 11, monodisperse suspension is formed, the main mode is significantly narrowed, and the size of aggregates is the smallest (of 97 nm). However, the average size of aggregates, calculated from the results of ten scans (within a measuring time of 120 s.) by Smoluchowski equation, changes symbate with the pH growth, and at pH 11 in 0.1-M solution of NaCl, it reaches 400 nm. This may indicate a dispersion exfoliation during measurement that is caused by coagulation with followed flotation/sedimentation process, or features of nanotube agglomerates to scatter the light.

As it was found earlier, nanotube suspensions are more stable in the alkaline media in comparison with those in the acidic media, and at the concentrations above 0.2 mg/ml, the zeta potential measurements became impossible because of low transparency of the suspensions. This conclusion relates to the suspension in KCl (0.01 M) [2]. At ten-time superiority in the electrolyte concentration of NaCl, the nanoparticle stabilizing charge is weak due to the adsorption of counterions which, in this case, are the anions of chlorine. Reducing the stability of suspensions of solid particles with increasing concentration of the electrolyte has been widely discussed in the literature and is associated with an excess of adsorbed counterions resulting in coagulation. This question for systems under study, as well as influence of the cation size, will be discussed below.

**Fig. 4:**
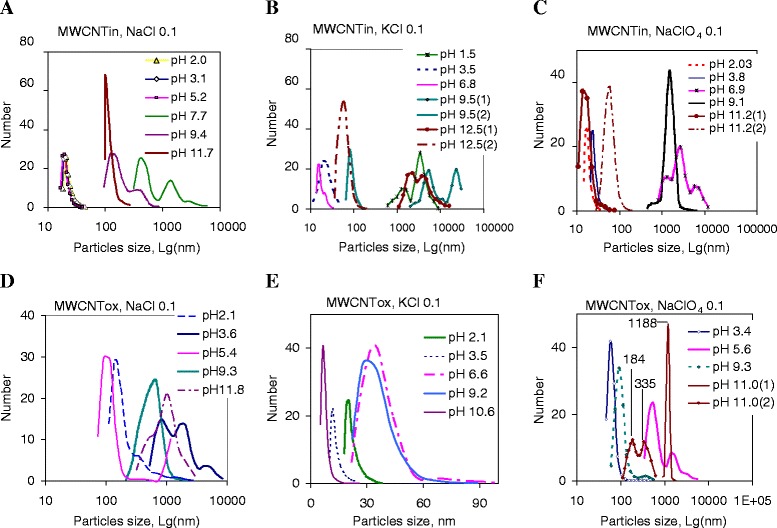
Particle size distribution in dispersions of pristine and oxidized MWCNTs. Figures show size distribution in dispersions of pristine (MWCNTin, **a**–**c**) and oxidized (MWCNTox, **d**–**f**) carbon nanotubes (dispersion concentration of 0.2 mg/ml) at different pH (pH range of 2–12) in NaCl (**a**, **d**), KCl (**b**, **e**), and NaClO_4_ (**c**, **f**) solutions with electrolyte concentration C = 0.1 mol/l

Considering the obtained data (Fig. 4a), one may conclude that during the transition to the alkaline region, an adsorption equilibrium which is necessary to stabilize the charge of the particles is disrupted possibly due to high concentrations of counter ions in the adsorbed layer, or coagulation of particles occurs due to the formation of oppositely charged areas at the interface nanotubes/fluids that provides self-discharge, or the main factor is the specific interaction of the nanotubes active sites with media components. The last aspect is determined by a presence of functional groups capable of dissociating or participating in the ion exchange process.

One should note the unusual features of nanotube dispersions obtained at pH 3.9, and 11. It also was marked for other carbon materials, namely, for a coal [4]. The authors attributed these features to process of surface functional group dissociation. As known, in acidic medium (below pH 3), a large number of H^+^ ions suppress the carboxyl group dissociation that promotes the electronegative charge decrease. In alkaline medium, dissociated H^+^ cations bind the excess OH^−^ anions that reduce the positive charge. At the same time, at pH < 3, carboxyl groups are protonated and provide positive surface charge. At pH 3–4, carboxylic groups dissociate that can promote dispersion stability. Another attractive point is pH 9. This is a region of phenolic group dissociation. One should also take into account the possibility of interaction of the carboxyl group with electrolyte cations to form a salt, which has an alkaline reaction and can react with chlorine anion. For carbon materials, a forming of surface chemical compounds is observed not only in adsorption of strong electrolytes polar mineral adsorbents but also in ion adsorption on carbon. For example, activated carbon is partially covered with oxides, and during the molecular adsorption behaves as a nonpolar adsorbent, but in solutions of strong electrolytes it is capable for surface chemical reactions.

Influence of surface reaction on the suspension stabilization is more substantial for dispersions of oxidized nanotubes. In case of additional surface coating by hydrolysis products and charged groups on dissociated sites, dispersion stabilization is complicated by partial surface recharging that leads to a discrete charge distribution and aggregation of particles. Also, a less basic residue, particularly phenolic, may also play a secondary role, since aromatic sites with strong acidic groups are known to affect nanotube electrokinetic properties [21]. It should be noted that a low content of oxygen-containing groups on pristine MWCNTs defines an increase in the agglomerate size. Diffusion of counterions inside agglomerates is complicated, and the degree of the functional group dissociation is reduced. Under such conditions, the EDL is formed in boundary layer of the aggregates and neutralization of the surface charge is realized at the lower concentration of the counter-ions. With a decrease in the nanotube concentration in volume due to partially sedimentation/flotation, the surface accessibility for more dispersed particles is increased and for the EDL formation, more counter-ions are required.

For dispersions of oxidized nanotubes prepared under the same conditions (solution NaCl, C = 0.1 mol/l) in the region of carboxylic groups dissociation (pH 3.5–5), the particle size distribution is narrow (Fig. 4d) with main mode position at 90–130 nm. The particle size distribution depends nonlinearly on the pH that caused by the availability of interfacial oxygen group capable not only of the ions absorbing but also for dissociation. In general, particles possess a surface charge, which occurs due to the dissociation of surface functional groups, the so-called Nernst potential. Of course, the degree of dissociation of the functional groups depends on the pH of the suspension; therefore, dispersion stabilization is pH dependent. In addition, concentrations of cationic ions increase by the dissociation of ionogenic groups or decrease by re-adsorption of some ions in solution on the solid particle surface. As a result of this, the electrical double layer around the particle becomes thinner or thicker, and thus the electrophoretic mobility of the solid particle changes. In the dispersion at pH 3.6, 9.7, and 11.8, bimodal particle size distribution is noted. The particle size at the transition to the alkaline pH region increases as it was observed for pristine nanotubes, but the narrowing of the main mode on the particle size distribution curve does not occur. In contrast, the basic modes for the subject systems are broadened, indicating the polydispersity of the systems.

### Effect of Electrolyte Ion Size on Particle Size in the Aqueous Dispersions of Nanotubes

The ability of ion to coagulate in colloidal solution depends upon both magnitude and sign of the charge. Coagulation ability of ions of the same valence may be expressed in lyotropic series in which the ions are arranged in decreasing or increasing their adsorption or coagulant activity. A specific sequence is related to ion energy and the hydration degree, which depend on the size and polarizability of the ion radius, i.e. deformability of the atom electron shells.

It was expected that monovalent ions under study are absorbed differently. The most active adsorption should be for ions with the largest radius. This is due to the fact that such ions have larger polarization degree and therefore are better attracted by polar group on the surface. Furthermore, the larger ion is less hydrated. And, it also enhances its adsorption, because the hydrate shell weakens the electrostatic attraction. Ions under study can be arranged in next series by adsorption activity:

$$ C{s}^{+} > {K}^{+} > N{a}^{+};\ Cl{O}^{4-}>C{l}^{-}. $$

Large ions can compress the electrical double layers surrounding the individual particles that increased charge of the particles and hence increased repulsive interaction between particles.

**Fig. 5:**
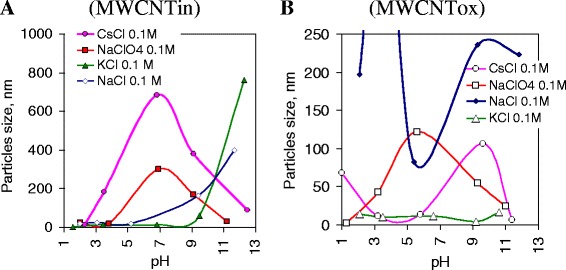
Particle size in dispersion of pristine and oxidized MWCNTs at different pH of electrolyte solutions. Figures 5 represents average particle (agglomerates) size that was obtained in dispersion of pristine (**a**) and oxidized (**b**) multiwalled carbon nanotubes dispersed in electrolytes with different cation (NaCl, KCl, CsCl) or anion (NaCl, NaClO_4_) size at concentration of 0.1 mol/l at different pH of solutions

Figure 5 shows the dependence of the average particle size in dispersion of pristine and oxidized MWCNTs in various electrolytes at different pH. For pristine MWCNTs, a similar character of the dependence for suspensions in the sodium and potassium chloride solutions was observed (Fig. 5a). Moreover, in a solution of potassium chloride, the relatively smaller particle size is observed up to pH 9, while an increase in agglomerate size for dispersions in sodium chloride is observed at pH 5. Other dependences were obtained for the dispersions of pristine MWCNTs in the CsCl and NaClO_4_ with the highest size of the cation and anion, respectively, among investigated electrolytes. This pair of curves has profile with extremum in the particle size of about pH 7, which may indicate the possibility of overcharging of the boundary surface of aggregates when crossing from acidic to alkaline medium. For dispersions with oxidized nanotubes (Fig. 5b), direct dependence of the coagulation ability of ions on their size is not marked. The smallest aggregate size in the whole pH range is observed for suspensions in KCl (Fig. 5b). However, the expected effect was partially obtained only for suspensions in sodium chloride (NaCl) solutions, the nanotube particle (aggregates) size in which was the largest. The curve’s profile for dispersions of oxidized nanotube in NaClO_4_ is identical to that for the pristine nanotube (Fig. 5a, b). The data obtained for pristine and modified MWCNTs suggest little influence of the surface oxygen-containing groups on a formation of the EDL in NaClO_4_ electrolyte.

For several suspensions, a prolongation of the dispersion stabilization was observed. This phenomenon has attracted attention because usual dispersions are coagulated with time. Namely, for the suspensions of pristine nanotubes in KCl (C = 0.1 mol/l) (Fig. 4b) in alkaline medium, significant narrowing of the particle size distribution curve is observed with a shift of main mode to lower particle sizes with keeping a high intensity. The particle size distribution diagram obtained indicates the hold of dispersed particle volume with a change in their hydrodynamic size to smaller. This effect is apparently related to the process of diffusion of the charged particles into inner area of aggregates with the subsequent formation of EDL on less available boundary layers or in pores causing agglomerate fragmentation.

**Fig. 6:**
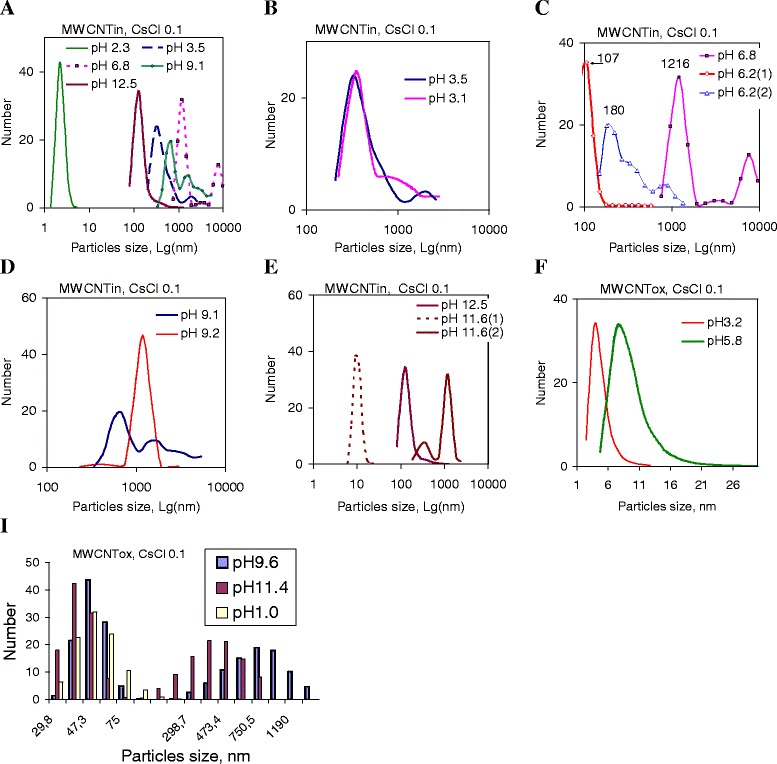
Particle size distribution in dispersions of pristine and oxidized MWCNTs in CsCl solution at different pH. Graphs show particle size distributions in dispersions of pristine (**a**–**e**) and oxidized MWCNTs (**f**, **i**) with concentration of 0.2 mg/ml in CsCl solution (C = 0.1 mol/l) at different pH. Markers 1 and 2 (**c**, **e**) correspond to measurements in the same suspension with a time (20 min). All dispersions were sonicated during 3 min at 22 kHz. The measurements were performed in 1 min after sonication

The stability of dispersions in the cesium chloride solution is considered separately, because unique stability of this dispersion at certain pH was observed. As it can be seen (Fig. 6a), the particle size distribution at different pH was relatively close. A feature of this system is very rapid coagulation of nanotubes at pH 2. The hydrodynamic size of the particles remaining in the solution is about 3–5 nm, which indicates their high mobility but does not allow assigning them to individual nanotubes with a charged surface. This effect for the pristine nanotubes may be due to lack of potential-determining ions, or, in the case of selective adsorption, of other ions with opposite sign. As a result, the charge and the potential of the surface of the particles decrease up closer to zero. At reducing the potential barrier, the so-called system’s neutralization coagulation occurs.

For suspension with pH close to pH of carboxyl group dissociation, the dispersion becomes stable (Fig. 6b). Figure 6b shows the results of independent experiments that were carried out with suspension pH 3.5 and had a very high reproducibility. At other pH, reproducibility was lower but the particle size distribution diagram profile (Fig. 6d) remained the same. Markers 1 and 2 (Fig 6c, e) represent the measurements in the same suspension with a time that allows us to track changes in the dispersion evolution. The narrow profile of the particle size distribution curves indicates the high homogeneity of suspensions. Two types of aggregates appeared in suspensions. Their formation depends on the time after sonication. For the whole range of pH, the prolongation of coagulation is noted.

For oxidized MWCNTs dispersed in the CsCl solution in acidic medium (pH 1, 3.2, and 5.8), the monomodal distribution of particles is observed (Fig. 6f, i), whereas the high pH transition (pH 9.6 and 11.4) promotes the bimodal particle distribution (Fig. 6f). For dispersions of oxidized nanotubes, each pH corresponds to a strict particle size distribution. An interesting fact is that at pH = 1, a particle size corresponds to one of the types of agglomerates formed in alkaline medium at pH 9.6 and 11.4. At a pH from 3 to 6, the smallest particle size is observed.

It is known that an increase in the electrolyte anion size leads to the polydispersity of the dispersed system. Studies have shown that for NaCl and NaClO_4_, bimodal particle distribution is observed in the same pH range (pH 7–9). With an increase in cation size, the pH range, where there is a bimodal distribution of particles, increases. Namely, for sodium chloride, the bimodal size distribution is detected in the pH range from 7 to 9, for potassium chloride, from pH 9 to pH 12, and for cesium chloride, pH 7–12. Additionally, for KCl, besides polydispersity in the strongly alkaline pH range 9–12, a bimodal distribution of particles was observed in a strongly acidic medium at pH 1.5.

### Effect of Electrolyte Concentration on Particle Size in Aqueous Dispersion of Carbon Nanotubes

Table 2 represents particle size in nanotube dispersion at different concentrations of electrolytes under study.

**Table 2:** The average particle size (*d*, nm) for the dispersion of pristine nanotubes (MWCNTin) in electrolyte solutions with different concentrations

Electrolytes	Concentration, mol/l	pH				
		2	3.5	~7	9.4	~12
NaCl	0.1	20.4	24.5	18.8	165.3	400.0
	0.01	458.2	84.8	20.3	208.0	14.8
	0.001	10.1	6.7	15.2	~3.0	14.3
KCl	0.1	3.2	15.2	14.4	62.3	763.0
	0.01	14.8	460.0	17.5	28.3	14.1
	0.001	17.8	120.9	52.8	866.3	208.3
CsCl	0.1	15.2	184.4	683.8	381.2	90.5
	0.01	33.3	199.9	82.9	211.7	8.3/165.0
	0.001	50.7	17.7	2.8	12.1	2.3
NaClO_4_	0.1	26.2	19.3	302.7	169.2	29.6
	0.01	173.1	315.2	46.3	35.2	78.8
	0.001	201.2	347.0	472.0	217.0	244.0

An increase in concentration usually leads to suspension coagulation, but for nanotube dispersions, a strict dependency is not observed. The dispersion stabilization is more affected by ion size and pH medium. For dispersions in NaCl and KCl, the change in particle size with an increase in the electrolyte concentration at pH 2 and 3.5 has the opposite character. In this case, the most significant differences were found at a concentration of 0.01 M indicating the predominant role of surface reactions.

Only for CsCl the particle size decreases with concentration decreasing, but at pH above 3. In acidic medium, the particle size was higher for electrolyte concentration of 0.001 M as for NaClO_4_ at the pH < 3.5.

Analysis an influence of the ion concentration on the particle size distribution in dispersions with the same pH shows the reversion of the dependences of the agglomerate size on the ion size. Namely, at pH 2, with increasing electrolyte concentration, the particle size decreases in a row: Na^+^ > ClO_4_^−^ > Cs^+^ gt; К^+^, and at pH 3.5—in the series: К^+^ > ClO_4_^−^ > Cs^+^ > Na^+^.

For pH 11, decreasing in electrolyte concentration causes the change in agglomerate size in order from К^+^ > Na^+^ > Cs^+^ > ClO_4_^−^ to ClO_4_^−^ > К^+^ > Na^+^ > Cs^+^. For anions under study at whole pH range with a decrease in electrolyte concentration, the largest size of the agglomerates is obtained in a solution of electrolyte with the largest anion size.

There are different models to understand stability and flocculation (aggregation) effect [22]. The simplest model of these phenomena is known as the DLVO (Deryaguin-Landau-Verwey-Overbeek) theory [23]. It simply states that the stability of the colloid is a balance between the attractive Van der Waals’ forces and the electrical repulsion due to the surface charge. If the zeta potential falls below a certain level, the colloid will aggregate due to the attractive forces. Conversely, a high zeta potential maintains a stable system. The point at which electrical and Van der Waals’ forces exactly balance can be identified with a specific electrolyte concentration, known as the critical flocculation concentration (CFC). As known, indifferent ions cause the zeta potential to continuously decline at high concentration, so we see a single CFC, and the colloid aggregates at all higher electrolyte concentrations. In contrast, specifically adsorbed ions cause charge reversal that may be sufficient to re-stabilize the colloid. In this case, we will see an upper and lower CFC, with a region of instability between them. Due to concurrence between ions in solutions under study, the strong correlation of aggregate size and electrolyte concentration was not observed. But, nanotube aggregation process is not a means that the surface has not charged. In order to obtain more information about nanotube boundary layer, the zeta potential was measured.

### Effect of Electrolyte Ion Size on Zeta Potential of Carbon Nanotubes

According to the literature data [24], as-produced carbon nanotubes have the isoelectric point (IEP) in the pH range of 5–8 and with increasing of pH, the zeta potential becomes more negative, because of adsorption of hydroxide ions on the MWCNT surface. Usually, after purification, the presence of acidic groups shifts IEP to the lowest pH values [25, 26]. As was shown in [27], purified nanotubes have small and positive zeta potential in the acidic medium with IEP in the pH range of 2–3. For tested herein, purified MWCNT IEP in KCl solution (0.1 M) was at pH about 2.5. After oxidation, the presence of oxygen-containing groups should promote a shift of the isoelectric point to values often below pH = 2 [28]. The oxidized MWCNTs usually exhibit negative charge in the whole pH range investigated, and the zeta potential values increased with pH growth that can be explained by dissociation of the hydroxyl groups that imparts the negative charge to the nanotubes surface.

**Fig. 7:**
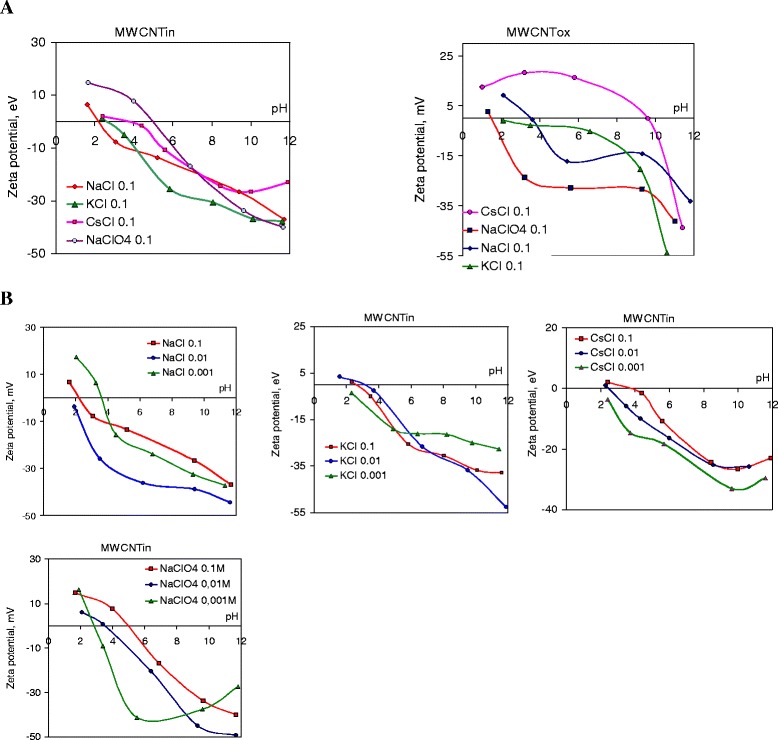
Zeta potential of pristine and oxidized MWCNTs in different electrolytes (**a**) and for dispersions of pristine nanotubes at different electrolyte concentration (**b**). Figure represents dependences of zeta potential on pH of electrolytes with different cation (NaCl, KCl, CsCl) and anion (NaCl, NaClO_4_) size. Concentration of nanotube dispersion is 0.2 mg/ml, electrolyte concentration is 0, 1, 0.01, and 0.001 mol/l, and sonication time for all dispersion was 3 min

The zeta potential of the pristine and oxidized nanotube particles was plotted as a function of pH of the dispersions in different electrolytes with electrolyte concentration 0.1 M (Fig. 7).

It can be observed in Fig. 7 that the zeta potential of pristine nanotubes in NaCl and KCl solutions is negative for the pH interval above 3.5 and its dependence on pH is in good agreement with the results published by other authors. With electrolyte ion size increase (Na^+^, K^+^, Cs^+^), isoelectric point (IEP) is shifted to a higher pH value from 2.1 to 4. Larger electrolyte anion size also promotes the shift of the IEP of pristine nanotubes from 2.1 in NaCl to 5 in NaClO_4_. An increase in zeta potential at high pH is mainly due to the interaction between OH ions and the positive sites of the nanotubes particle which are rendered neutral or negative by the adsorption of the hydroxyl ions. At the lowest pH values, the zeta potential indicates lower negative values owing to the H^+^ adsorption on the negative charge of the particle surface. The recharging of the nanotube zeta potential in CsCl at isoelectric point at pH = 4 indicates an adsorption of cations in the acidic pH range, and the same intensive sorption of anions at the transition to greater pH. This may be due to the formation of EDL with a more complex structure as a consequence of the passage of the surface recharge.

Considering the possible reasons of recharging the surface of nanotubes in the electrolyte solution by Cs^+^ ions should take into account the following. The EDL occurs on the surface of the nanotubes due to the presence of the charged oxygen-containing groups (ionization of carboxylic and phenolic groups, etc.) and structural defects (sp^3^-hybridization of the carbon bonds in benzene rings), and the nanotube surface in aqueous dispersion is negatively charged. In the presence of larger and weakly hydrated cations Cs^+^, which are strongly electrostatically attracted to the surface, the charge of the nanotube surface is neutralized. Then, a specific over-equivalent adsorption of Cs^+^ ions is carried out leading to the appearance of excess positive charge on the nanotube surface. Surface charge obtained is compensated by negative counterions from solution (namely, Cl^−^), and on the surface, a new EDL with the positive internal ions and negative counterions in the outer layer appeared [28].

The character of the curve (Fig. 7) in pH range of 3–9 for dispersion of NaCl and NaClO_4_ is similar to those of the plots obtained in [27, 29] for oxidized nanotubes. The low potential values (in the range from −10 to −35 mV) demonstrate that the MWCNTs are less sensitive to protons (H^+^) and hydroxyl anions (OH^−^) [30]. It is usual when the functional groups are primary ketones, aldehydes, alcohols, or esters, because these groups are electrically neutral and do not dissociate in the pH range of 2–12.

The magnitude of the zeta potential for nanotubes under study (Fig. 7a) testifies that oxidized nanotubes contain mainly groups with low hydrolysis or ionization ability; it can be alcohol and ester groups. For dispersion of nanotubes after oxidation (MWCNTox), the zeta potential should become more negative with isoelectric point (IEP) shifting to lower pH values, in comparison to the dispersions of pristine MWCNTs. However, this relationship is realized only for the dispersion in the potassium chloride and in NaClO_4_. Furthermore, for oxidized nanotube dispersions in NaClO_4_, a change in the structure of EDL is greater with shifting of the IEP from 1.5 (for pristine MWCNTin) to pH = 5 for MWCNTox (Fig. 7a). For dispersion in sodium chloride and in cesium chloride with an increase in the concentration of surface oxygen-containing groups, the IEP is shifted to larger pH and the zeta potential magnitude decreases in the whole pH range. In the dispersion of oxidized nanotubes in CsCl below pH 9.5, the zeta potential is positive, indicating the preferential adsorption of the cesium cations on the nanotube surface. For suspensions in electrolytes with different sizes of anion (NaCl and NaClO_4_), with the anion size increase, the IEP shifted to lower pH, indicating a decrease of the thickness of the EDL.

### Effect of Electrolyte Concentration on Zeta Potential of Carbon Nanotubes

As outlined above, the zeta potential and physical stability should decrease at increasing electrolyte concentration. In the case of nanotubes, increasing the electrolyte concentration (Fig. 7b) in general reduces the magnitude of zeta potential with offset to a positive charge that may not necessarily result in coagulation because steric factor also impacts on the dispersion stability. The thickness of the diffuse double layer is also affected by the concentration of the exchangeable cations. Generally [31], it has been reported that at equivalent electrolyte concentrations, monovalent cations in exchange positions yield thicker diffuse double layers in antibate relation with ion size (or symbate relation with ion hydration shell). This phenomenon is due to the tendency of ions to diffuse away from the colloidal particle surface (van Olphen, 1977). The change in the zeta potential of the pristine nanotube dispersion with decreasing of the electrolyte concentration is nonlinear (Fig. 7b). Mainly for the tested systems, an increase in the concentration from 0.1 to 0.01 M results in the formation of the EDL with a higher zeta potential value (more negative). However, further electrolyte dilution shifts the zeta potential value to the positive range. Thus, with increasing in CsCl concentrations closely to pH = 2, zeta potential changes from negative charge to positive. For other electrolytes, a similar trend is detected. The transfer of charge significantly reduces the stability of dispersions.

Over the whole concentration range for electrolytes under study, the zeta potential values indicate a negative charge on the particles at pH above 6. As for zeta potential evolution in a strongly acidic pH, in alkaline medium, the magnitudes of zeta potential at electrolyte concentration for 0.1 and 0.001 M are close. The increase in the electrolyte concentration from 0.001 to 0.01 M increases the zeta potential electronegativity. So, increasing the concentration of ions with maximum hydration shell, but forming the thickest diffusion layer among the studied solutions, does not lead to a significant reduction in zeta potential and aggregation of the system, as seen in [3]. The positively charged metal ion neutralizes the surface charge on the surfaces of MWCNTs and compresses the double layer, resulting in the reduction of electrostatic repulsion between nanotubes. Thus, the zeta potential depends on the concentration of the electrolyte solution while the surface charge is determined by the specific adsorption properties of the ions. This may explain the different effects of ions of the same valence and stability of coagulation of colloidal solutions.

### Effect of Electrolyte Ion Size and Concentration on Surface Charge Density of Pristine and Oxidized MWCNTs

In general, particles possess a surface charge, which occurs due to the dissociation of surface functional groups, the so-called Nernst potential. Of course, the degree of dissociation of the functional groups depends on the pH of the suspension; therefore, the surface charge is pH dependent.

**Fig. 8:**
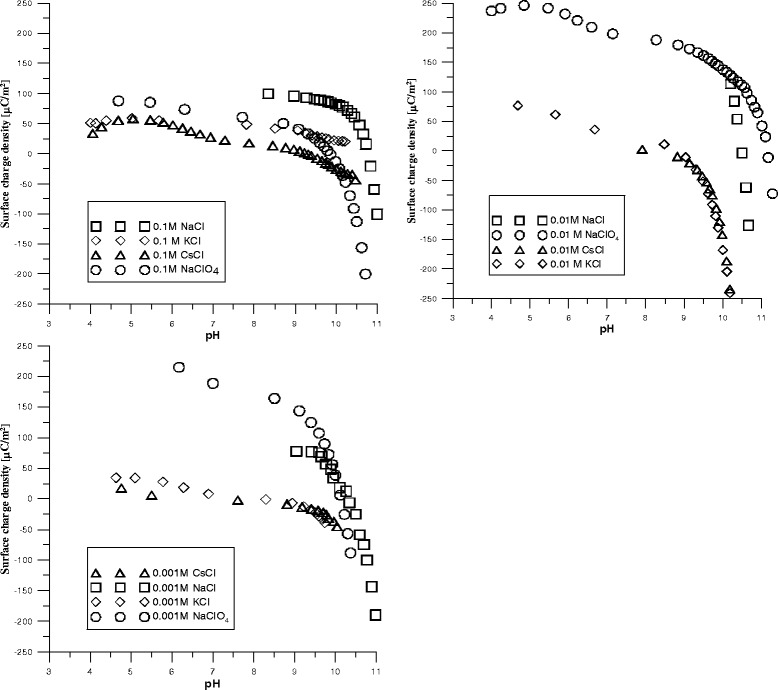
Surface charge density at different pH for dispersion of pristine MWCNT. Figures present dependences of surface charge of pristine MWCNTs on pH in solutions of monovalent cations (NaCl, KCl, CsCl) and anions (NaCl and NaClO_4_) with concentration 0.1, 0.01, and 0.001 M

The effects of monovalent cations under study on the charge density are not similar. Obtained results suggest that the potential is determined by positively charged surface sites. With the change in the size of the cation, the surface charge is reduced to other sign, as among cations Na^+^, K^+^, and Cs^+^, at pH 9, surface charge changed from 100 to −100 μC/m^2^. During the transition in the alkaline medium above pH 9 (region of OH-group dissociation), the charge increases significantly with the negative sign (Fig. 8).

It should be noted that with increasing concentration of the electrolyte, the charge is changing from negative to positive. In all solutions, the point of zero charge and the sign of the surface charge changed with the electrolyte concentration. The same features have the dispersion of pristine nanotubes in NaClO_4_ solutions, where upon an increase of the electrolyte concentration, the magnitude of the charge is changed and the point of zero charge slightly shifts to the high pH (Fig. 7b). Hence, the charge on the surface is determined by adsorbed ions, and only in solution of potassium chlorine at lowest concentration (0.001 M) adsorption centres with different charge magnitude that are characteristic for the surface of pristine nanotubes are distinguished. With the KCl concentration increase, the surface charge becomes mostly positive. With increasing electrolyte concentration, the surface charge will be compensated at a lower distance from the particle surface, which means the potential drops faster and the diffuse layer is thinner.

The increase in surface charge when the concentration of the monovalent electrolyte decreases significantly is caused by adsorption of anions (Cl^−^) on the surface. This adsorption would help to increase the surface charge. On the other hand, it was found that when the concentration of KCl was increased in the liquid medium around the particles, the net negative charge (at the Stern layer) of nanotubes was decreased, as a consequence of the packing restriction ions. When the monovalent ions were compared, the charge density of the particles had the greatest value in NaCl solution. This could be related to the hydration radius of ions. Na^+^ ions could enter into the electrical double layer only to a small extent owing to the large radius of this (hydrated) ion.

**Fig. 9:**
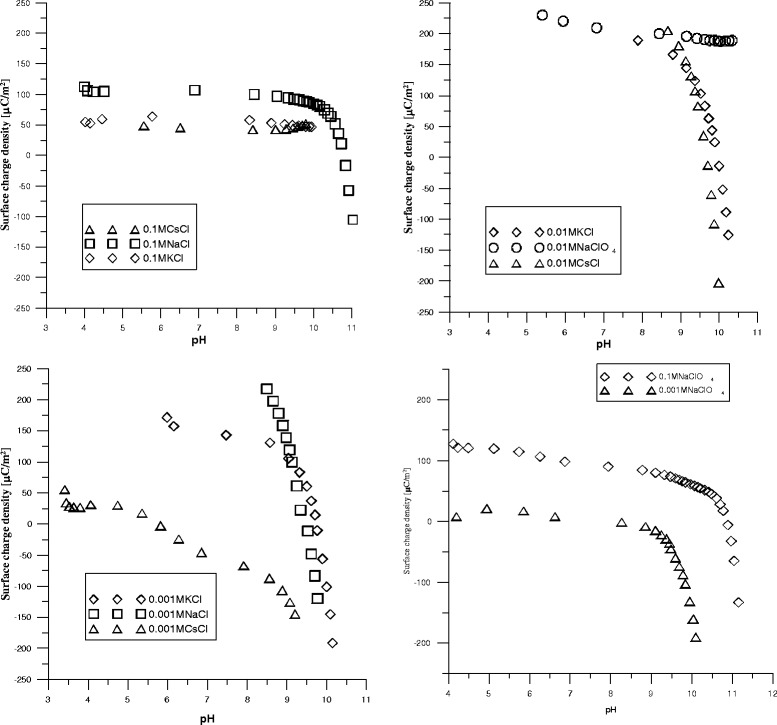
Surface charge density at different pH for dispersion of oxidized MWCNT. Figures present dependences of surface charge of oxidized MWCNTs on pH in solutions of monovalent cations (NaCl, KCl, CsCl) and anions (NaCl and NaClO_4_) with concentration 0.1, 0.01, and 0.001 M

After nanotube oxidation, surface should have more charged sites but significant increasing in surface charge density was not detected (Fig. 9).

As for dispersions of pristine nanotubes, the surface charge of oxidized MWCNTox in KCl solution changes sign at electrolyte concentration and decreases from positive to negative. Concentration of NaCl had significant effect on the value of surface charge and had less effect on a position of zero charge point. For dispersions in CsCl, the electrolyte concentration increase results in shifts of surface charge to positive value (Fig. 9). For these electrolytes, the surface should be rendered more positive with an increase in the concentration of the electrolytes. The zeta potential values of nanotube particles become less negative with an increase in concentration from 10^−2^ M to 10^−1^ M due to the compression of the double layer by the presence of electrolyte. The counter ions are accumulated in the electrical double layer (EDL), and they render the particle surface less negative. Yet, up to 10^−3^ M, the surface charge became more negative. Hydrolysis causes the replacement of M^+^ by H^+^ for concentrations of MCl (M = Na^+^, K^+^, Cs^+^) of 10^−3^ M. This ion-exchange mechanism occurs in two steps: (1) a rapid exchange between hydrogen ions in solution and the exchangeable cations of the nanotubes, according to the schematic equation: M-MWCNT + H_2_O → ← H-MWCNT + MOH and (2) a slow penetration of the adsorbed H^+^ ions into the lattice. This attack by protons gives rise to a decomposition of the surface layer and a release of exchangeable ions. On the other hand, the decomposition process just described occurs at a considerably slower rate at the higher electrolyte concentration. Therefore, it will have little effect under conditions of high ionic strength.

According to [28], at any MCl concentration, the magnitude of the surface charge should increase with the electrolytes in the order Na^+^ > K^+^ > Cs^+^. Equilibrium constants for cation exchange, hydration free energies, hydration ion size, and enthalpies of exchange for various exchangeable cations show that the strength with which M^+^ ions are held to the surface of the nanoparticles is in the order Cs^+^ > K^+^ > Na^+^. Namely, the degree of hydration of these cations affects either their dissociation from the Stern layer or the thickness of this layer. It was concluded that the more strongly hydrated monovalent cations (K^+^, Na^+^) are bound more weakly to the surface and therefore give rise to greater potentials (Horikawa et al., 1988). In contrast, the less hydrated monovalent cations (Cs^+^) are bound more strongly to the surface and give rise to relatively small potentials.

Furthermore, the size of the hydrated ion in the Stern layer should influence the thickness of the layer and thus the magnitude of the potential. The Cs^+^ ions have small hydrated ionic radii and a small distance of closest approach to the surface relative to other monovalent ions. Thus, Cs^+^ ions could enter the Stern layer easily, and charge became less negative in comparison to other electrolytes.

## Conclusions

It was found that in the dispersion of the pristine nanotubes in NaCl (C = 0.1 mol/l and pH 1.89) after sonication, the particle size distribution changes in time: nanotube agglomerates were formed in about 2–5 min after sonication and monodispersion is transformed into polydispersion with a low stability of agglomerates. For dispersion of pristine nanotubes in NaCl 0.1 mol/l, better stability was achieved at pH below 3 in spite of low zeta potential value. Upon the transition to the alkaline pH region, the particle size increases for both oxidized and pristine nanotubes.

Among the electrolytes under study, the smallest aggregate size in the whole pH range was observed for suspensions in KCl, and the largest one—in sodium chloride at concentration 0.1 mol/l. In solution of CsCl, the smallest particle size is observed at pH from 3 to 6.

The nanotube dispersion stabilization is more affected by ion size and pH medium than the electrolyte concentration. The data obtained confirms the predominant role of surface reactions. At the pH 2 with increasing electrolyte concentration, the particle size in the dispersion of the initial nanotubes decreases in a row Na^+^ > ClO_4_^−^ > Cs^+^ > К^+^, and at pH 3.5, in the series К^+^ > ClO_4_^−^ > Cs^+^ > Na^+^. For pH 11, decreasing in electrolyte concentration changes the agglomerate size in order from К^+^ > Na^+^ > Cs^+^ > ClO_4_^−^ to ClO_4_^−^ > К^+^ > Na^+^ > Cs^+^. For anions under study at whole pH range with a decrease in electrolyte concentration, the largest size of the agglomerates is obtained in a solution of electrolyte with the largest anion size.

In the presence of larger and weakly hydrated cations Cs^+^, the charge of the nanotube surface is neutralized, and then specific over-equivalent adsorption of Cs^+^ ions leads to the appearance of excess positive charge on the nanotube surface.

For suspensions in electrolytes with different sizes of anion (NaCl and NaClO_4_), with the anion size increase, the IEP shifted to lower pH, indicating a decrease in the thickness of the EDL.

The increase in the electrolyte concentration in general reduces the magnitude of zeta potential with offset to a positive charge that does not result in coagulation. With the change in size of the cation, the surface charge is reduced to other sign, as among cations Na^+^, K^+^, and Cs^+^ at pH 9, surface charge changed from 100 to −100 μC/m^2^. Upon the transition into the alkaline medium above pH 9 (region of OH group dissociation), the charge increases significantly with the negative sign. With increasing the concentration of the electrolyte, the nanotube surface charge changes from negative to positive. Obtained results show that the strength with which M^+^ ions are held to the surface of the nanoparticles is in the order Cs^+^ > K^+^ > Na^+^. As was found, the most stable dispersion of nanotubes is achieved in CsCl electrolyte solution at less negative charge of the surface.

## Abbreviations

IEP: isoelectric point
EDL: electrical double layer
MWCNTs: multiwalled carbon nanotubes
PDI: potential determining ion
TEM: transmission electron microscopy
FT-IR: Fourier transform infrared spectroscopy
DLVO: Deryaguin-Landau-Verwey-Overbeek theory
CFC: critical flocculation concentration
